
JOURNAL INFORMATION
==============================
NLM Title Abbreviation: ScientificWorldJournal
Journal ID: ScientificWorldJournal
NLM Journal ID: 101131163
Journal ID: TSWJ

The Scientific World Journal

ISSN: 2356-6140
EISSN: 1537-744X
Publisher: Wiley

ARTICLE INFORMATION
==============================
PMCID: PMC6084410
PMID: 30147548
DOI: 10.1100/tsw.2001.112
Article ID: 812784
Article version: 1
Subjects: Short Report

Controlled Neuronal Death during Aging Might Be an Attractive Therapeutic Target for Preventing Alzheimer's Disease

Schmitz Christoph *
Korr Hubert
Department of Anatomy and Cell Biology, RWTH University of Aachen, Pauwelsstrasse / Wendlingweg 2, D-52057 Aachen, Germany
*Christoph Schmitz: cschmitz@post.klinikum.rwth-aachen.de
Publication date: 2001
Electronic publication date: 2001 Aug 8
Volume: 1
First page: 72
Last page: 72
Copyright: Copyright © 2001 Christoph Schmitz and Hubert Korr.
Copyright year: 2001
License: This is an open access article distributed under the Creative Commons Attribution License, which permits unrestricted use, distribution, and reproduction in any medium, provided the original work is properly cited.
License URL: https://creativecommons.org/licenses/by/3.0/

Funding: Deutsche Forschungsgemeinschaft Ko 715/3-1 Deutsche Forschungsgemeinschaft SCHM 1561/2-1

==============================
Miami *Nature Biotechnology* Short Reports
*TheScientificWorld* (2001) 1(S3), 72SR
ISSN 1532-2246; DOI 10.1100/TSW.2001.112

### CONTROLLED NEURONAL DEATH DURING AGING MIGHT BE AN ATTRACTIVE THERAPEUTIC TARGET FOR PREVENTING ALZHEIMER'S DISEASE

Christoph Schmitz* and Hubert Korr

Department of Anatomy and Cell Biology, RWTH University of Aachen, Pauwelsstrasse / Wendlingweg 2, D-52057 Aachen, Germany
*cschmitz@post.klinikum.rwth-aachen.de

**INTRODUCTION.** On the one hand, the molecular basis of the selective neuronal vulnerability in Alzheimer's disease (AD) is still largely unknown despite intense research over the last decades. On the other hand, it was recently found that the aging mouse brain shows - among others - parallels with human neurodegenerative disorders at the transcriptional level (1). Since both a decline of nuclear (n) DNA repair capacity and mitochondrial (mt) dysfunction have been demonstrated to be important aspects of the pathogenesis of AD, we have studied the extent of nDNA repair and mtDNA synthesis in the aging mouse brain quantitatively, specific for different cell types and in situ.

**METHOD.** Untreated male NMRI-mice were kept under specific pathogen free conditions. At the age of 2, 6, 9, 15, 20, 25 and 30 months, four mice each were investigated by using quantitative autoradiography after injection of [$^3$H]-thymidine. The analyses were focused on the extent of spontaneous overall nDNA repair by measuring nuclear labeling representing unscheduled DNA synthesis (UDS) as well as on the extent of mtDNA synthesis by measuring cytoplasmic labeling (see 2 for methodical details).

**RESULTS.** For all investigated types of neurons an age-related decrease of the extent of mtDNA synthesis was found. By contrast, only distinct types of neurons (i.e., projection neurons of, e.g., cerebral cortex, hippocampus and thalamus) showed a decrease of UDS. Most strikingly, there was a highly positive correlation between the pattern of neurons in the mouse brain showing the age-related decrease of UDS (pattern X) and the pattern of neurons in the human brain which - according to the literature - are affected by the formation of neurofibrillary tangles in AD (pattern Y). Moreover, there was a highly negative correlation between pattern X and the pattern of neurons that - according to the literature - show an age-related neuron loss in both rodent and human brain (pattern Z). With respect to the recent discussion in the literature regarding the validity of results concerning age-related neuron loss due to methodological shortcomings, only studies in line with the current state-of-the-art in stereology (3) were considered here.

**DISCUSSION.** There are several lines of evidence in the literature that an age-related decline of nDNA repair is connected with accumulation of unrepaired nDNA damage, mitochondrial dysfunction, increased amount of reactive oxygen species (ROS) in the corresponding cells and subsequent cellular dysfunction. In distinct types of neurons in the aging mouse brain, however, this seems to be prevented by physiological, age-related cell death (2). Based on the data shown here we have hypothesized that in the human brain - as in the mouse brain - there is a group of neurons suffering from age-related decrease of nDNA repair, accumulation of unrepaired nDNA damage, mitochondrial dysfunction and increased amount of ROS (2). This group of neurons is identical to that group of neurons being affected by the formation of neurofibrillary tangles in AD. Furthermore, an accumulation of ROS, a decline of nDNA repair capacity and mitochondrial dysfunction have been shown to be important aspects of the pathogenesis of AD. Therefore, mimicking the physiological, age-related cell death (see above) by therapeutic interventions for all types of projection neurons in the aging human brain (i.e., controlled neuronal death during aging) might be a very attractive therapeutic target for preventing Alzheimer's disease.

**ACKNOWLEDGEMENT.** This work was supported by Deutsche Forschungsgemeinschaft Grants Ko 715/3-1 and SCHM 1561/2-1.

**REFERENCES.**

1. Lee, C.K., Weindruch, R., and Prolla, T.A. (2000) Nat. Genet. 25, 294-297
2. Schmitz, C., Materne, S., and Korr, H. (1999) J. Alz. Dis. 1, 387-407
3. Schmitz, C. and Hof, P.R. (2000) J. Chem. Neuroanat. 20, 93-114
